# The autocrine role of proteoglycan-4 (PRG4) in modulating osteoarthritic synoviocyte proliferation and expression of matrix degrading enzymes

**DOI:** 10.1186/s13075-017-1301-5

**Published:** 2017-05-08

**Authors:** Ali Alquraini, Maha Jamal, Ling Zhang, Tannin Schmidt, Gregory D. Jay, Khaled A. Elsaid

**Affiliations:** 1 Department of Pharmaceutical Sciences, MCPHS University, Boston, MA USA; 2 Department of Emergency Medicine, Rhode Island Hospital, Providence, RI USA; 3 Faculty of Kinesiology and Schulich School of Engineering, University of Calgary, Calgary, AB Canada; 4 Department of Biomedical Engineering, Brown University, Providence, RI USA; 5 Department of Biomedical and Pharmaceutical Sciences, Chapman University School of Pharmacy, Rinker Health Sciences Campus, 9401 Jeronimo Road, Irvine, CA 92618 USA; 6 School of Pharmacy, Albaha University, Albaha, Saudi Arabia

**Keywords:** Lubricin, Proteoglycan-4, CD44, Osteoarthritis, Fibroblast-like synoviocytes

## Abstract

**Background:**

Lubricin/proteoglycan 4 (PRG4) is a mucinous glycoprotein secreted by synovial fibroblasts and superficial zone chondrocytes. Recently, we showed that recombinant human PRG4 (rhPRG4) is a putative ligand for CD44 receptor. rhPRG4-CD44 interaction inhibits cytokine-induced rheumatoid arthritis synoviocyte proliferation. The objective of this study is to decipher the autocrine function of PRG4 in regulating osteoarthritic synoviocyte proliferation and expression of catabolic and pro-inflammatory mediators under basal and interleukin-1 beta (IL-1β)-stimulated conditions.

**Methods:**

Cytosolic and nuclear levels of nuclear factor kappa B (NFκB) p50 and p65 subunits in *Prg4* ^*+/+*^ and *Prg4* ^*-/-*^ synoviocytes were studied using western blot. Nuclear translocation of p50 and p65 proteins in osteoarthritis (OA) fibroblast-like synoviocytes (FLS) in response to IL-1β stimulation in the absence or presence of rhPRG4 was studied using DNA binding assays. OA synoviocyte (5000 cells per well) proliferation following IL-1β (20 ng/ml) treatment in the absence or presence of rhPRG4 (50–200 μg/ml) over 48 hours was determined using a colorimetric assay. Gene expression of matrix metalloproteinases (*MMPs*), tissue inhibitor of metallproteinases-1 (*TIMP-1*), *TIMP-2*, *IL-1*β, *IL-6*, *IL-8*, *TNF-*α, cycloxygenae-2 (*COX2*) and *PRG4* in unstimulated and IL-1β (1 ng/ml)-stimulated OA synoviocytes, in the presence or absence of rhPRG4 (100 and 200 μg/ml), was studied following incubation for 24 hours.

**Results:**

*Prg4* ^*-/-*^ synoviocytes contained higher nuclear p50 and p65 levels compared to *Prg4* ^*+/+*^ synoviocytes (*p* < 0.05). rhPRG4 (100 μg/ml) reduced p50 and p65 nuclear levels in *Prg4* ^*+/+*^ and *Prg4* ^-/-^ synoviocytes (*p* < 0.001). Similarly, rhPRG4 (200 μg/ml) inhibited NFκB translocation and cell proliferation in OA synoviocytes in a CD44-dependent manner (*p* < 0.001) via inhibition of IκBα phosphorylation. IL-1β reduced *PRG4* expression in OA synoviocytes and rhPRG4 (100 μg/ml) treatment reversed this effect (*p* < 0.001). rhPRG4 (200 μg/ml) reduced basal gene expression of *MMP-1*, *MMP-3*, *MMP-13*, *IL-6*, *IL-8*, and *PRG4* in OA synoviocytes, while increasing *TIMP-2* and cycloxygenase-2 (*COX2*) expression (*p* < 0.001). rhPRG4 (200 μg/ml) reduced IL-1β induction of *MMP-1*, *MMP-3*, *MMP-9*, *MMP-13*, *IL-6*, *IL-8*, and *COX2* expression in a CD44-dependent manner (*p* < 0.001).

**Conclusion:**

PRG4 plays an important anti-inflammatory role in regulating OA synoviocyte proliferation and reduces basal and IL-1β-stimulated expression of catabolic mediators. Exogenous rhPRG4 autoregulates native PRG4 expression in OA synoviocytes.

## Background

Lubricin/proteoglycan-4 (PRG4) is a glycoprotein secreted by synovial fibroblasts and superficial zone chondrocytes [1–3]. The importance of PRG4 to joint hemostasis is evidenced in the loss-of-function mutations in the *Prg4* gene in the autosomal recessive disease, camptodactylyl-arthropathy-coxa vara pericarditis (CACP) syndrome, characterized by juvenile-onset arthropathy [4, 5]. The joints of *Prg4* knockout (*Prg4* ^-/-^) mice display progressive synovial hyperplasia, cartilage surface fibrillations, and chondrocyte apoptosis, which may not be completely reversed by PRG4 re-expression [6–8]. Therapeutically, the recombinant and native forms of PRG4 have been shown to exhibit a disease-modifying effect in pre-clinical osteoarthritis (OA) models [9–14].

A biological role for recombinant human PRG4 (rhPRG4) was recently reported. rhPRG4 was shown to compete with hyaluronan for binding to the CD44 receptor resulting in downstream inhibition of nuclear factor kappa B (NFκB) nuclear translocation in synoviocytes from patients with rheumatoid arthritis (RA) [15]. Furthermore, rhPRG4 inhibits cytokine-induced proliferation of murine *Prg4* ^-/-^ synovial fibroblasts and human synovial fibroblasts derived from patients with RA [15]. rhPRG4 has also been shown to interact with toll-like receptors 2 and 4 (TLR2 and TLR4) and may fulfill an anti-inflammatory role [15–17].

CD44 is a major cell surface receptor, with various isoforms generated by alternative splicing and glycosylations, which possesses the ability to bind different ligands and exert different biological functions in inflammation and cancer [18, 19]. Increased expression of CD44 was reported in experimental OA and in cartilage and synovia of patients with different severities of OA [20–23]. Given the emerging evidence on the role of PRG4 in regulating synoviocyte proliferation in response to inflammatory stimuli, we sought to decipher the autocrine role of PRG4 in regulating OA synoviocyte proliferation and expression of matrix metalloproteinases (MMPs), tissue inhibitors of matrix metalloproteinases (TIMPs), aggrecanase-1 (a disintegrin and metalloproteinase with thrombospondin motifs 4 (ADAMTS4)), aggrecanase-2 (ADAMTS5), and pro-inflammatory and chemotactic cytokines. Additionally, we studied the regulation of *PRG4* gene expression in OA fibroblast-like synoviocytes (FLS) in response to interleukin-1 beta (IL-1β) and rhPRG4 treatments. We hypothesized that PRG4 is an important autocrine modulator of synovial cells, mediated by its interaction with CD44.

## Methods

### NFκB p50 and p65 cytosolic and nuclear levels in *Prg4*^+/+^ and *Prg4*^-/-^ synoviocytes and impact of rhPRG4 treatment

Synovial tissues were harvested from *Prg4* ^+/+^ and *Prg4* ^-/-^ male mice 8–10 weeks old and synoviocytes were isolated as described [15]. *Prg4* ^+/+^ and *Prg4* ^-/-^ synoviocytes were cultured in T-25 flasks until confluence. *Prg4* ^*+/+*^ and *Prg4* ^*-/-*^ synoviocytes were treated with rhPRG4 (100 μg/ml) [24], CD44 monoclonal antibody (CD44 Ab) (1.25 μg/ml) (Abcam, USA) or a combination of rhPRG4 and CD44 Ab for 48 hours. Nuclear and cytosolic cell extractions were performed using NE-PER nuclear and cytoplasmic extraction reagents kit (Thermo Fisher Scientific, USA). A total of 20 μg of protein was loaded into the wells of 4–12% Bis-Tris gels (Thermo Fisher Scientific) followed by gel electrophoresis and western blotting. Membranes were blocked using 5% nonfat dry milk in PBS-T for 1 hour at room temperature. Subsequently, membranes were incubated with anti-NFκB p50 antibody (Santa Cruz Biotechnology, USA) (1:1000 dilution) or anti-NFκB p65 antibody (Abcam) (1:1000 dilution) overnight at 4 °C. Membranes were also incubated with anti-Lamin B1 antibody (Abcam) (nuclear loading control) or anti β-Actin antibody (Cell signaling Technologies, USA) (cytosolic loading control). After washing three times with PBS-T, membranes were incubated with IRDye 800CW goat anti-rabbit secondary antibody (1:10,000 dilution) (LI-COR, USA) at room temperature for 1 hour. After washing three times with PBS-T, membranes were imaged using Odyssey CLx imaging system (LI-COR). Densitometry analysis was performed using Image J software. Data are presented as the densitometry ratio of p50 or p65 and either Lamin B1 or β-Actin in the same sample. Data are presented as the average ± standard deviation of three independent experiments.

### IL-1β induced NFκB p50 and p65 nuclear translocation in OA FLS, and IL-1β induced cell proliferation and impact of rhPRG4 treatment

OA FLS (Cell Applications, USA) were harvested from patients undergoing total knee replacement. Cells were used between the third and sixth passages in all experiments. OA FLS (300,000 cells per well) were stimulated with IL-1β (20 ng/ml; R&D Systems, USA) for 6 hours at 37 °C in the presence or absence of rhPRG4 (100 μg/ml), rhPRG4 (200 μg/ml), rhPRG4 (200 μg/ml) + CD44 Ab (2.5 μg/ml; Abcam) or CD44 Ab (2.5 μg/ml). Cells were harvested and nuclear extraction was performed using a commercially available kit (Thermo Fisher Scientific). Total protein was quantified, and 3 μg of nuclear extract from each experimental group was used. The p50 and p65 proteins were detected in the nuclear extract using NFκB DNA binding assay kits (Abcam). Data are presented as p50 or p65 nuclear levels normalized to untreated controls. Data are presented as the average ± the standard deviation of four independent experiments, each with duplicate wells per group.

OA FLS were seeded in T-25 flasks at 1,000,000 cells in DMEM + 10% FBS for 48 hours. The total volume was 4 ml. After 48 hours, medium was removed and replaced with DMEM + 1% FBS. Cells were incubated for 6 hours in the absence or presence of rhPRG4 (100 and 200 μg/ml) and/or CD44 Ab (2.5 μg/ml; pre-treatment for 2 hours prior to rhPRG4 treatment) followed by treatment with IL-1β (1 ng/ml) for 30 minutes.

Subsequently, medium was removed and cells were rinsed twice with ice cold PBS (1 ml). Protein extraction reagent (M-PER, Thermo Fisher Scientific) supplemented with protease and phosphatase cocktail inhibitor (1:100 dilution; Thermo Fisher Scientific) and EDTA (1:100 dilution; Thermo Fisher Scientific) was added (300 μl per flask) and cells were collected. Protein samples (20 μg/ml; 40 μl per well) were loaded in 10% PAGE pre-cast gels (Bio-Rad, USA). Following gel electrophoresis and transfer, membranes were blocked using 5% bovine serum albumin (BSA) for 1 hour at room temperature. Membranes were probed for phosphorylated inhibitor kappa B alpha (p-IκBα) or total IκBα using commercially available rabbit antibodies (Abcam). Antibodies were diluted 1:1000 in 5% BSA and incubated with membranes overnight at 4 °C. After washing with Tris-buffered saline Tween 20 (TBS-T), membranes were incubated with horseradish peroxidase (HRP)-conjugated goat anti-rabbit (1:5000) for 1 hour at room temperature. Membranes were also probed for β-Actin using a commercially available β-Actin antibody (1: 10,000) (Abcam). Protein bands were developed using SuperSignal West Pico PLUS chemiluminescent substrate (Thermo Fisher Scientific) and visualized using C-Digit Target (LI-COR, USA).

OA FLS in 96-well plates (5000 cells per well) in DMEM supplemented with 1% FBS and 1 mM pyruvate were stimulated with IL-1β (20 ng/ml) for 48 hours at 37 °C in the absence or presence of rhPRG4 at a final concentration of 50, 100, or 200 μg/ml. The total volume in each well was 200 μl. Cell proliferation was determined using the Cell Titer 96 AQueous one solution cell proliferation assay (MTS; Promega, USA) and absorbance at 490 nm was measured. In a separate set of experiments, rhPRG4 (200 μg/ml) treatment was performed in the absence or presence of a CD44 Ab (2.5 μg/ml; Abcam) and cell proliferation was determined as described previously. Data are presented as fold of OA FLS proliferation normalized to untreated controls. Data are presented as the average ± standard deviation of four independent experiments, each with triplicate wells per group.

### Modulation of PRG4 secretion by OA FLS and RA FLS and impact of rhPRG4 on PRG4 gene expression in unstimulated and IL-1β stimulated OA synoviocytes

OA FLS and RA FLS (Cell Applications) were grown in DMEM + 10% FBS and used between the third and sixth passages: 20,000 cells per well were plated in sterile 96-well plates and incubated at 37 °C for 48 hours to allow cell attachment. The total volume per well was 200 μl. The medium was changed to DMEM + 1% FBS and cells were treated with IL-1β (1 ng/ml), tumor necrosis factor alpha (TNF-α) (1 ng/ml; R&D Systems), or transforming growth factor beta (TGF-β) (1 ng/ml; R&D Systems) for 72 hours. Subsequently, medium supernatants were collected and PRG4 concentrations were determined using an inhibition ELISA as previously described [16]. PRG4 concentrations were normalized to cell density, determined colorimetrically using the Cell Titer 96 AQueous one solution cell proliferation assay (MTS; Promega) and the 490 nm absorbance was measured. Data are presented as media PRG4 content normalized to cell density. Data are presented as the average ± standard deviation of four independent experiments, each with duplicate wells per group.

OA FLS (250,000 cells per well) were treated with IL-1β (1 ng/ml) in the absence or presence of rhPRG4 (100 or 200 μg/ml) for 24 hours followed by RNA extraction using Triazol reagent (Thermo Fisher Scientific), and RNA concentrations were determined using a NanoDrop ND-2000 spectrophotometer (NanoDrop Technologies, USA). cDNA was synthesized using Transcriptor First Strand cDNA Synthesis Kit (Roche, USA). Quantitative PCR (qPCR) was performed on Applied Biosystems StepOnePlus Real-Time PCR System (Thermo Fisher Scientific) using TaqMan Fast Advanced Master Mix (Life Technologies, USA). The cycle threshold (Ct) value of PRG4 (Hs00981633_m1; Thermo Fisher Scientific) was normalized to the Ct value of GAPDH (Hs02758991_g1; Thermo Fisher Scientific) in the same sample, and the relative expression was calculated using the 2^-ΔΔCt^ method [25]. In another set of experiments, rhPRG4 (100 and 200 μg/ml) was incubated with OA FLS for 24 hours followed by RNA isolation, cDNA synthesis, and PRG4 qPCR as described above. Data are presented as fold PRG4 gene expression compared to untreated control. Data are presented as the average plus on minus standard deviation of four independent experiments with duplicate wells per treatment.

### Modulation of IL-1β-induced OA FLS proliferation by OA SF and the role of synovial fluid PRG4

Synovial fluid (SF) samples were collected from patients with OA (*n* = 5) (Articular Engineering, USA) following knee replacement surgery [16]. Four of the OA patients were female and the median age of the group was 65 years. OA SF was pooled from the five patients. PRG4 immunoprecipitation was conducted as described previously [16]. PRG4 depletion was confirmed by assaying pooled OA SF for PRG4 levels using an inhibition ELISA as previously described [16].

IL-1β-induced OA FLS proliferation was conducted as described previously in the absence or presence of pooled OA SF or PRG4-immunoprecipitated OA SF at 20 μl or 40 μl per well (corresponding to 10% or 20% dilution). Cell proliferation was determined using the Cell Titer 96 AQueous one solution cell proliferation assay (MTS; Promega) and the 490 nm absorbance was measured. Data are presented as fold OA FLS proliferation compared to untreated controls. Data are presented as the average ± standard deviation of four independent experiments, each with duplicate wells per treatment.

### PRG4 knockdown in OA FLS and proliferation of PRG4-silenced OA FLS

OA FLS (250,000 cells per well) were treated with a pre-validated PRG4 small interfering RNA (siRNA) (Thermo Fisher Scientific) (25 pmol per well) or a non-targeted negative control (NC) siRNA (25pmoles) (Thermo Fisher Scientific) for 48 hours. Transfection was performed using Lipofectamine RNAiMAX (Thermo Fisher Scientific) per manufacturer’s recommendations. To confirm PRG4 knockdown, *PRG4* gene expression was determined in PRG4 siRNA and NC siRNA-treated OA FLS as described previously and compared to *PRG4* gene expression in untreated control OA FLS. IL-1β stimulation of OA FLS proliferation in control, NC siRNA, and PRG4 siRNA-treated cells was performed as described previously. IL-1β stimulation was performed for 24 hours and cell proliferation was measured as described previously. OA FLS proliferation of the different experimental groups was normalized to untreated control cells. Data are presented as the average ± standard deviation of three independent experiments with duplicate wells per treatment.

### Impact of rhPRG4 treatment on target gene expression in unstimulated and IL-1β-stimulated OA FLS

OA FLS (250,000 cells per well) were treated with rhPRG4 (100 and 200 μg/ml) for 24 hours followed by RNA isolation, cDNA synthesis and qPCR was performed as described previously. Target genes included *MMP-1* (Hs00899658_m1), *MMP-2* (Hs00234422_m1), *MMP-3* (Hs00968305_m1), *MMP-9* (Hs00234579_m1), *MMP-13* (Hs00233992_m1), *TIMP-1* (Hs00171558_m1), *TIMP-2* (Hs00234278_m1), *ADAMTS4* (Hs00192708_m1), *ADAMTS5* (Hs00199841_m1), *IL-1*β (Hs00174097_m1), *IL-6* (Hs00985639_m1), *IL-8* (Hs00174103_m1), *TNF-*α (Hs00174128_m1), and cycloxygenase-2 (*COX2*) (HS00153133_m1). All primers and probes are commercially available (Thermo Fisher Scientific). The Ct values of target genes were calculated and normalized to the Ct value of *GAPDH* in the same sample and relative gene expression was calculated as described previously. Data are presented as fold expression of target genes in rhPRG4-treated OA FLS compared to expression in untreated control OA FLS. Data are presented as the average ± standard error of the mean (SEM) of four independent experiments.

OA FLS (250,000 cells per well) were treated with IL-1β (1 ng/ml) in the absence or presence of rhPRG4 (100 and 200 μg/ml) for 24 hours followed by RNA isolation, cDNA synthesis, and qPCR as described previously. Target genes included *MMP-1*, *MMP-2*, *MMP-3*, *MMP-9*, *MMP-13*, *TIMP-1*, *TIMP-2*, *ADAMTS4*, *ADAMTS5*, *IL-1*β, *IL-6*, *IL-8*, *TNF-*α and *COX2*. The Ct values of target genes were calculated and normalized to the Ct value of GAPDH in the same sample, and relative gene expression was calculated as described previously. Data are presented as fold expression of target genes in the IL-1β group, IL-1β + rhPRG4 (100 μg/ml) or IL-1β + rhPRG4 (200 μg/ml)-treated OA FLS groups, compared to expression in the control group. Data are presented as the average ± SEM of four independent experiments.

In another set of experiments, CD44 neutralization was achieved by pre-incubating OA FLS (250,000 cells per well) with CD44 Ab (2.5 μg/ml; Abcam) for 2 hours followed by treating OA FLS with IL-1β + rhPRG4 (200 μg/ml) for 24 hours followed by RNA isolation, cDNA, and qPCR as described previously. Data are presented as the average ± SEM of four independent experiments.

### Statistical analyses

The nuclear NFκB p50 and p65 protein levels in OA FLS treatments were normalized to untreated control levels. The average of the absorbance values in untreated control cells across the four independent experiments was used to normalize absorbance values in the different experimental groups across the four independent experiments. A similar approach was used to present OA FLS proliferation. Statistical analyses of gene expression data was performed using ΔCt values (C_t_ target gene-C_t_ *GAPDH*) for each gene of interest in each experimental group and data were graphically presented as fold expression relative to untreated controls using the 2^-ΔΔCt^ method. Continuous variables were initially tested for normality and equal variances. Variables that satisfied both assumptions were tested for statistical significance using Student’s *t* test or analysis of variance (ANOVA) with Tukey’s post-hoc test for comparisons of two groups and more than two groups, respectively. Variables that did not satisfy the normality assumption were tested using the Mann-Whitney *U* test or ANOVA on the ranks. The level of statistical significance was set at α = 0.05.

## Results

### Cytosolic and nuclear NFκB p50 and p65 proteins in *Prg4*^*+/+*^ and *Prg4*^*-/-*^ synoviocytes and impact of rhPRG4 treatment

**Fig. 1:**
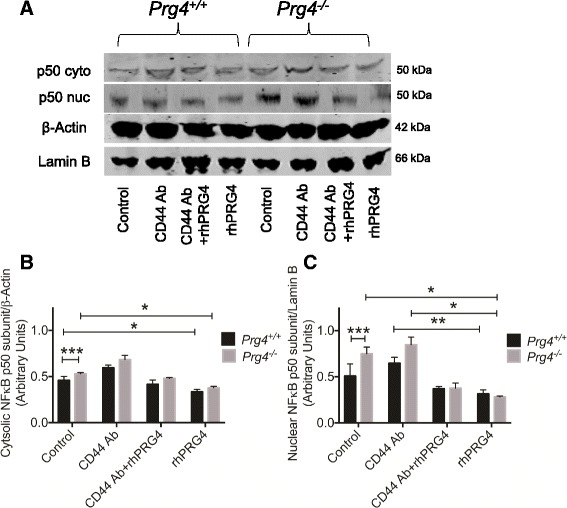
The impact of recombinant human proteoglycan 4 (*rhPRG4*) treatment on cytosolic and nuclear levels of nuclear factor kappa B (*NF*κ*B*) p50 subunit in *Prg4* ^*+/+*^ and *Prg4* ^*-/-*^ synoviocytes; *p < 0.001, **p < 0.01, ***p < 0.05. **a** Representative western blot showing cytosolic and nuclear p50 protein in *Prg4* ^*+/+*^ and *Prg4* ^*-/-*^ synoviocytes in control, CD44 antibody (*CD44 Ab*)-treated, CD44 Ab + rhPRG4-treated and rhPRG4-treated cells. Lamin B1 was used as a nuclear loading control and β-Actin was used as a cytosolic loading control. **b** Semi-quantitative densitometry analysis of normalized cytosolic p50 protein in untreated and rhPRG4-treated *Prg4* ^*+/+*^ and *Prg4* ^*-/-*^ synoviocytes. *Prg4* ^*-/-*^ synoviocytes had higher cytosolic p50 compared to *Prg4* ^+/+^ synoviocytes. rhPRG4 (100 μg/ml) reduced cytosolic p50 levels in *Prg4* ^+/+^ and *Prg4* ^-/-^ synoviocytes. Co-incubation with CD44 Ab (1.25 μg/ml) did not alter the effect of rhPRG4. Data are presented as mean ± standard deviation of three independent experiments. **c** Semi-quantitative densitometry analysis of normalized nuclear p50 protein in untreated and rhPRG4-treated *Prg4* ^*+/+*^ and *Prg4* ^*-/-*^ synoviocytes. *Prg4* ^*-/-*^ synoviocytes had higher nuclear p50 compared to *Prg4* ^+/+^ synoviocytes. rhPRG4 (100 μg/ml) reduced nuclear p50 levels in *Prg4* ^+/+^ and *Prg4* ^-/-^ synoviocytes. Co-incubation with CD44 Ab (1.25 μg/ml) did not alter the effect of rhPRG4. Data are presented as mean ± standard deviation of three independent experiments

Cytosolic and nuclear p50 in rhPRG4 and CD44 antibody (Ab)-treated and untreated synoviocytes is shown in Fig. 1. A representative blot of p50 protein, cytosolic and nuclear loading controls is shown in Fig. 1a. We have observed stronger p50 bands in the cytosolic and nuclear fractions of *Prg4* ^*-/-*^ synoviocytes compared to *Prg4* ^*+/+*^ cells. Consistently, rhPRG4 treatment reduced cytosolic and nuclear p50 levels in both *Prg4* ^*+/+*^ and *Prg4* ^*-/-*^ synoviocytes. Semi-quantitative analysis of normalized cytosolic and nuclear p50 band intensities from the different experimental groups is presented in Fig. 1b and c. Cytosolic and nuclear p50 subunit levels were significantly higher in *Prg4* ^*-/-*^ synoviocytes compared to *Prg4* ^*+/+*^ synoviocytes (*p* < 0.05). There was no significant difference in cytosolic and nuclear levels of p50 between untreated control and CD44 Ab-treated *Prg4* ^*+/+*^ synoviocytes or *Prg4* ^*-/-*^ synoviocytes. rhPRG4 treatment significantly reduced cytosolic and nuclear p50 levels compared to untreated control *Prg4* ^*+/+*^ and *Prg4* ^*-/-*^ synoviocytes (*p* < 0.001). A trend towards an increase in cytosolic and nuclear p50 levels in the CD44 Ab + rhPRG4 group compared to the rhPRG4 alone group was observed although it was not statistically significant (*p* > 0.05).

**Fig. 2:**
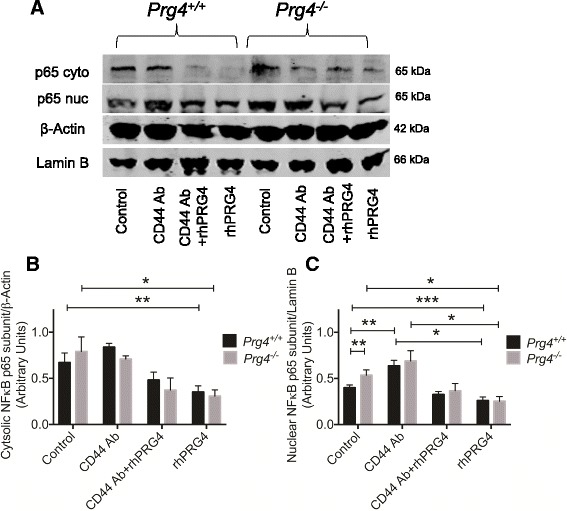
The impact of recombinant human proteoglycan 4 (*rhPRG4*) treatment on cytosolic and nuclear levels of nuclear factor kappa B (*NF*κ*B*) p65 subunit in *Prg4* ^*+/+*^ and *Prg4* ^*-/-*^ synoviocytes; *p < 0.001, **p < 0.01, ***p < 0.05. **a** Representative western blot showing cytosolic and nuclear p65 protein in *Prg4* ^*+/+*^ and *Prg4* ^*-/-*^ synoviocytes in control, CD44 antibody (*CD44 Ab*)-treated, CD44 Ab + rhPRG-treated, and rhPRG4-treated cells. Lamin B1 was used as a nuclear loading control and β-Actin was used as a cytosolic loading control. **b** Semi-quantitative densitometry analysis of normalized cytosolic p65 protein in untreated and rhPRG4-treated *Prg4* ^*+/+*^ and *Prg4* ^*-/-*^ synoviocytes. rhPRG4 (100 μg/ml) reduced cytosolic p65 levels in *Prg4* ^+/+^ and *Prg4* ^-/-^ synoviocytes. Co-incubation with CD44 Ab (1.25 μg/ml) did not alter the effect of rhPRG4. Data are presented as mean ± standard deviation of three independent experiments. **c** Semi-quantitative densitometry analysis of normalized nuclear p65 protein in untreated and rhPRG4-treated *Prg4* ^*+/+*^ and *Prg4* ^*-/-*^ synoviocytes. *Prg4* ^*-/-*^ synoviocytes had higher nuclear p65 compared to *Prg4* ^+/+^ synoviocytes. rhPRG4 (100 μg/ml) reduced nuclear p65 levels in *Prg4* ^+/+^ and *Prg4* ^-/-^ synoviocytes. Co-incubation with CD44 Ab (1.25 μg/ml) did not alter the effect of rhPRG4. Data are presented as mean ± standard deviation of three independent experiments

Cytosolic and nuclear p65 protein in rhPRG4 and CD44 Ab-treated and untreated synoviocytes is shown in Fig. 2. p65 protein was detectable in the cytosolic and nuclear fractions in *Prg4* ^*+/+*^ and *Prg4* ^*-/-*^ synoviocytes (Fig. 2a). rhPRG4 treatment consistently reduced p65 staining intensity in the cytosolic and nuclear fractions in both genotypes. Semi-quantitative analysis of normalized cytosolic and nuclear p50 band intensities from the different experimental groups is presented in Fig. 2b and c. Nuclear p65 subunit levels were significantly higher in *Prg4* ^*-/-*^ synoviocytes compared to *Prg4* ^*+/+*^ synoviocytes (*p* < 0.01). CD44 Ab-treated *Prg4* ^*-/-*^ synoviocytes had significantly higher nuclear p65 levels compared to untreated controls (*p* < 0.01). rhPRG4 treatment significantly reduced cytosolic and nuclear p65 levels compared to untreated control *Prg4* ^*+/+*^ and *Prg4* ^*-/-*^ synoviocytes (*p* < 0.001). Similarly, a trend towards an increase in cytosolic and nuclear p65 levels in the CD44 Ab + rhPRG4 group compared to the rhPRG4 alone group was observed although it was not statistically significant (*p* > 0.05).

### rhPRG4 inhibited IL-1β-stimulated NFκB p50 and p65 nuclear translocation in OA FLS mediated by inhibition of IκBα phosphorylation in a CD44-dependent manner

**Fig. 3:**
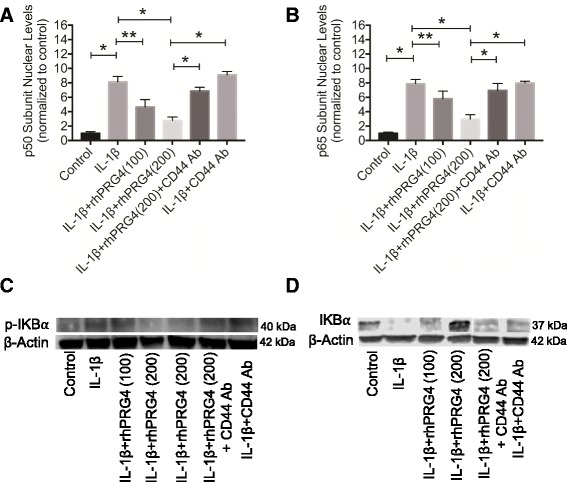
The impact of recombinant human proteoglycan 4 (*rhPRG4*) treatment on interleukin-1 beta (*IL-1*β)-induced nuclear factor kappa b (*NF*κ*B*) p50 and p65 nuclear translocation, inhibitor kappa B alpha (*I*κ*B*α) phosphorylation and degradation. Data are presented as mean ± standard deviation of four independent experiments; *p < 0.001, **p < 0.01. **a** Impact of rhPRG4 treatment on IL-1β-induced p50 protein nuclear translocation in ostoarthritis fibroblast-like synoviocytes (OA FLS). IL1β treatment induced p50 protein nuclear translocation. rhPRG4 treatment (100 μg/ml and 200 μg/ml) inhibited IL-1β-induced p50 protein nuclear translocation. Co-incubation with a CD44 neutralizing monoclonal antibody (CD44 Ab) abolished the effect of rhPRG4 treatment. CD44 Ab treatment alone did not alter p50 nuclear translocation. **b** Impact of rhPRG4 treatment on IL-1β-induced p65 protein nuclear translocation in OA FLS. IL1β treatment induced p65 protein nuclear translocation. rhPRG4 treatment (100 μg/ml and 200 μg/ml) inhibited IL-1β-induced p65 protein nuclear translocation. Co-incubation with a CD44 neutralizing monoclonal antibody (CD44 Ab) abolished the effect of rhPRG4 treatment. CD44 Ab treatment alone did not alter p65 nuclear translocation. **c** Representative western blot of cytosolic phosphorylated inhibitor kappa B alpha (*p-I*κ*B*α) in untreated and IL-1β-treated OA synoviocytes in the absence or presence of rhPRG4 and/or CD44 Ab. rhPRG4 (200 μg/ml) reduced p-IκBα levels in a CD44-dependent manner. **d** Representative western blot of cytosolic total inhibitor kappa B alpha (*I*κ*B*α) in untreated and IL-1β-treated OA synoviocytes in the absence or presence of rhPRG4 and/or CD44 Ab. rhPRG4 (200 μg/ml) increased IκBα levels in a CD44-dependent manner

IL-1β treatment significantly increased NFκB p50 and p65 nuclear levels in OA FLS compared to untreated OA FLS (*p* < 0.001) (Fig. 3a and b). rhPRG4 (100 μg/ml) treatment significantly reduced IL-1β-induced p50 and p65 nuclear translocation (*p* < 0.01). Similarly, rhPRG4 (200 μg/ml) treatment significantly reduced IL-1β induced p50 and p65 nuclear translocation (*p* < 0.001). There was no significant difference in the p50 and p65 nuclear level in the IL-1β + CD44 Ab group and the IL-1β group. NFκB p50 and p65 nuclear levels in the IL-1β + rhPRG4 (200 μg/ml) + CD44 Ab group were significantly higher than corresponding levels in the IL-1β + rhPRG4 (200 μg/ml) group (*p* < 0.001) and were not significantly different from nuclear levels in the IL-1β + CD44 Ab group. The ability of rhPRG4 to lower NFκB nuclear levels was inhibited by co-incubation with CD44 Ab, which showed no difference from the IL-1β group.

A representative p-IκBα western blot in control and IL-1β-treated OA FLS in the presence or absence of rhPRG4 with or without CD44 Ab co-treatment is shown in Fig. 3c. IL-1β treatment increased p-IκBα levels compared to untreated control. rhPRG4 (200 μg/ml) treatment reduced IκBα phosphorylation compared to IL-1β alone. This effect was reversed by a CD44 Ab. We did not observe a difference in p-IκBα level between the IL-1β group and the IL-1β + CD44 Ab group. A representative total IκBα western blot in control and IL-1β-treated OA FLS in the presence or absence of rhPRG4 with or without CD44 Ab co-treatment is shown in Fig. 3d. IL-1β treatment reduced total IκBα. Total IκBα was higher with IL-1β + rhPRG4 (200 μg/ml) compared to IL-1β alone. Co-incubation with a CD44 Ab abolished the effect of rhPRG4 on total IκBα following IL-1β stimulation. We did not observe a difference in total IκBα content between the IL-1β group and the IL-1β + CD44 Ab group.

### rhPRG4 inhibited IL-1β-stimulated proliferation in OA FLS in a CD44-dependent manner

**Fig. 4:**
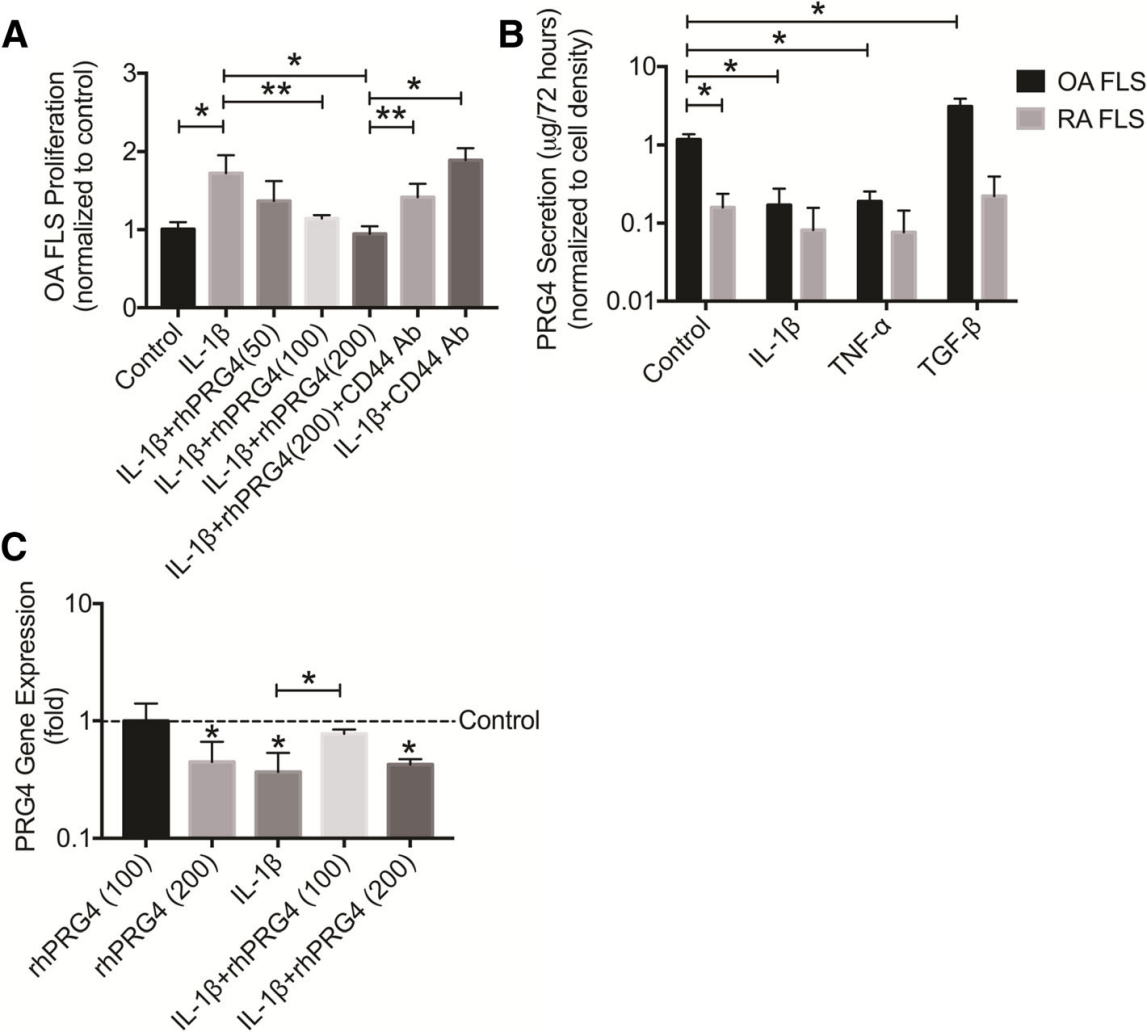
The impact of recombinant human proteoglycan 4 (*rhPRG4*) treatment on interleukin-1 beta (*IL-1*β)-induced osteoarthritis fibroblast-like synoviocytes (*OA FLS*) proliferation and regulation of proteoglycan-4 (*PRG4*) gene expression and production. Data are presented as the average ± standard deviation of four independent experiments; *p < 0.001, **p < 0.05. **a** Impact of rhPRG4 treatment (50, 100, and 200 μg/ml) on IL-1β-induced proliferation of OA FLS over 48 hours. IL-1β induced OA FLS proliferation. rhPRG4 (100 and 200 μg/ml) reduced IL-1β-induced OA FLS proliferation. Co-treatment with a CD44 neutralizing monoclonal antibody (*CD44 Ab*) abolished the effect of rhPRG4 treatment. CD44 Ab treatment alone did not alter IL-1β-induced OA FLS proliferation. **b** Regulation of PRG4 production by cytokines in OA FLS and rheumatoid arthritis FLS (RA FLS). OA FLS produces significantly higher PRG4 protein compared to RA FLS. IL-1β and TNF-α reduce PRG4 production by OA FLS while transforming growth factor beta (*TGF*-β) increases PRG4 production by OA FLS. IL-1β, TNF-α, and TGF-β did not alter PRG4 production by RA FLS. **c** IL-1β downregulated *PRG4* gene expression in OA FLS. *PRG4* expression in the IL-1 β + rhPRG4(100 μg/ml) group was higher than *PRG4* expression in the IL-1β alone group and was not different from *PRG4* expression in control cells. *PRG4* expression in the IL-1β + rhPRG4(200 μg/ml) group was lower than *PRG4* expression in the control and in the IL-1β + rhPRG4(100 μg/ml) group. rhPRG4 (200 μg/ml) treatment reduced basal *PRG4* gene expression in OA FLS compared to control. Data are presented as fold change compared to untreated control OA FLS

IL-1β induced OA FLS proliferation over 48 hours (*p* < 0.001) (Fig. 4a). rhPRG4 (50 μg/ml) treatment did not reduce IL-1β-induced OA FLS proliferation. In contrast, rhPRG4 (100 and 200 μg/ml) treatments significantly reduced IL-1β-induced OA FLS proliferation (*p* < 0.01; *p* < 0.001). CD44 Ab and rhPRG4 co-treatment resulted in significantly greater OA FLS proliferation compared to rhPRG4 treatment (*p* < 0.01) and was not significantly different from OA FLS proliferation in the IL1β alone group. Finally, there was no significant difference in OA FLS proliferation between the IL-1β + CD44 Ab group and the IL-1β group.

### Modulation of PRG4 production in OA FLS and RA FLS and effect of rhPRG4

Basal PRG4 production in OA FLS was significantly higher than PRG4 production by RA FLS (*p* < 0.001) (Fig. 4b). IL-1β and TNF-α reduced PRG4 production by OA FLS compared to control untreated cells (*p* < 0.001). TGF-β enhanced PRG4 production by OA FLS compared to control untreated cells (*p* < 0.001). IL-1β, TNF-α, and TGF-β did not significantly alter PRG4 production by RA FLS compared to untreated cells.

rhPRG4 (100 μg/ml) treatment did not alter basal *PRG4* gene expression in OA FLS (Fig. 4c). In contrast, rhPRG4 (200 μg/ml) significantly reduced *PRG4* gene expression in OA FLS (*p* < 0.001). IL-1β reduced *PRG4* gene expression in OA FLS (*p* < 0.001). *PRG4* expression in the IL-1β + rhPRG4 (100 μg/ml) group was significantly higher than *PRG4* expression in IL-1β alone (*p* < 0.001) and the IL-1β + rhPRG4 (200 μg/ml) group (*p* < 0.01). Alternatively, there was no significant difference in *PRG4* gene expression between the IL1β group and the IL1β + rhPRG4 (200 μg/ml) group. Finally, *PRG4* gene expression in the IL-1β + rhPRG4 (200 μg/ml) treatment was significantly lower than PRG4 expression in untreated control OA FLS (*p* < 0.001).

### SF PRG4 depletion and PRG4 downregulation promotes OA FLS proliferation

**Fig. 5:**
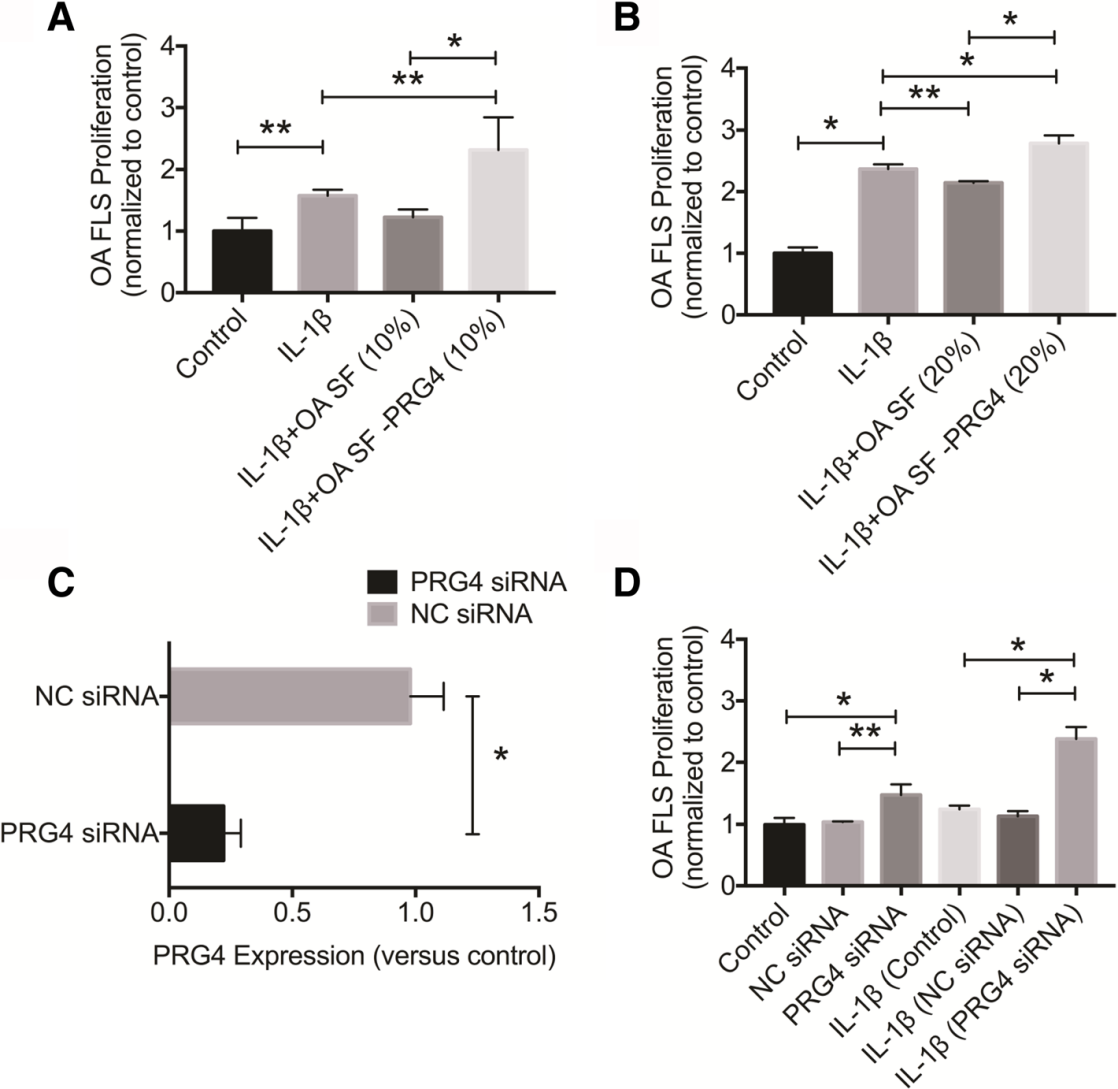
The role of proteoglycan 4 (*PRG4*) resident in synovial fluids from patients with osteoarthritis (*OA*) and produced by OA fibroblast-like synoviocytes (*OA FLS*) in modulating interleukin-1 beta (*IL-1*β)-induced OA FLS proliferation. Data are presented as mean ± standard deviation of at least three independent experiments. OA FLS proliferation of the different experimental groups was normalized to untreated control cells; *p < 0.001, **p < 0.05. **a** Impact of synovial fluid PRG4 depletion on cellular proliferation in an in vitro model of IL-1β-induced OA FLS proliferation over 48 hours. IL-1β induced OA FLS proliferation and OA SF (10%) did not alter the effect of IL-1β on OA FLS. PRG4-depleted OA SF (*OA SF – PRG4*) enhanced the proliferative effect of IL-1β on OA FLS. **b** Impact of synovial fluid PRG4 depletion on cellular proliferation in an in vitro model of IL-1β-induced OA FLS proliferation over 48 hours. IL-1β induced OA FLS proliferation and OA SF (20%) significantly reduced IL-1β-induced OA FLS proliferation. PRG4 depletion reversed the effect of OA SF and enhanced the proliferative effect of IL-1β on OA FLS. **c** *PRG4* gene knockdown in OA FLS using PRG4 small interfering RNA (*siRNA*). Treatment with a non-targeting negative control (*NC*) siRNA over a 48-hour period did not alter PRG4 gene expression. PRG4 siRNA treatment reduced endogenous PRG4 expression in OA FLS. Data are normalized to PRG4 expression in untreated control OA FLS. **d** Impact of PRG4 knockdown on OA FLS proliferation under unstimulated and IL-1β stimulated conditions. PRG4 knockdown enhanced OA FLS proliferation under basal conditions compared to NC treatment over 24 hours. PRG4 knockdown enhanced IL-1β-induced OA FLS proliferation compared to NC treatment over 24 hours

Mean PRG4 concentration in OA SF after PRG4 immunoprecipitation was 25.11 ± 3.18 μg/ml, compared to 280.43 ± 14.76 μg/ml in OA SF with no PRG4 immunoprecipitation. OA SF treatment at 10% dilution did not significantly alter IL-1β-induced OA FLS proliferation (Fig. 5a). PRG4-deficient OA SF (10% dilution) significantly increased IL-1β-induced OA FLS proliferation compared to OA SF (10% dilution) (*p* < 0.001) or no SF treatment (*p* < 0.01). OA SF (20% dilution) significantly reduced IL-1β-induced OA FLS proliferation (*p* < 0.01) (Fig. 5b). PRG4-deficient OA SF (20% dilution) significantly increased IL-1β induced OA FLS proliferation compared to OA SF (20% dilution) or no SF treatment (*p* < 0.001). NC siRNA treatment did not significantly alter *PRG4* gene expression in OA FLS compared to untreated OA FLS (Fig. 5c). In contrast, PRG4 siRNA treatment resulted in a significant reduction in *PRG4* gene expression, approximating an 80% reduction (*p* < 0.001) compared to untreated or NC siRNA-treated OA FLS. PRG4 siRNA-treated OA FLS displayed significantly higher basal cell proliferation compared to NC siRNA-treated OA FLS (*p* < 0.01) or untreated OA FLS (*p* < 0.001) over 24 hours (Fig. 5d). Similarly, and following IL-1β stimulation, PRG4 siRNA-treated OA FLS had significantly higher proliferation compared to NC siRNA-treated OA FLS or non-silenced control OA FLS over 24 hours (*p* < 0.001).

### Expression of OA-associated catabolic enzymes is affected by rhPRG4 in unstimulated and IL-1β-stimulated OA FLS

**Fig. 6:**
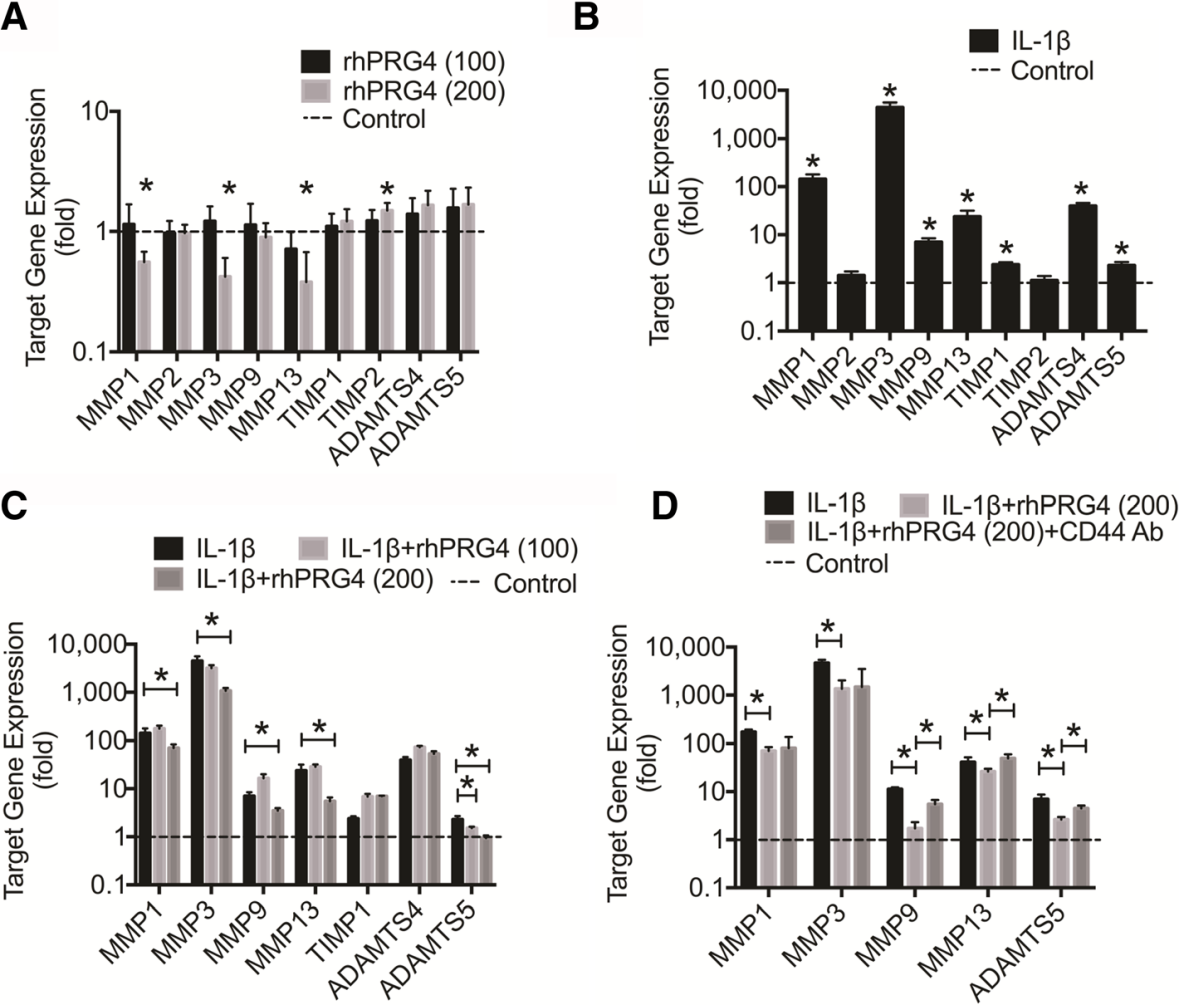
The impact of recombinant human proteoglycan-4 (*rhPRG4*) treatment on basal and IL-1β-induced gene expression of matrix metalloproteinases (*MMPs*), aggrecanases 1 and 2 (*ADAMTS4* and *ADAMTS5*), and tissue inhibitor of metalloproteinases (*TIMPs*) in OA fibroblast-like synoviocytes (*OA FLS*). Data are presented as the average ± standard error of the mean (SEM) of four independent experiments. Data are represented as fold change compared to untreated control OA FLS; *p < 0.001. **a** Effect of rhPRG4 treatment on basal gene expression in OA FLS. rhPRG4 (200 μg/ml) reduced expression of *MMP1*, *MMP3*, and *MMP13*. rhPRG4 (200 μg/ml) increased expression of *TIMP-2*. **b** Impact of IL-1β treatment on catabolic enzyme gene expression in OA FLS. IL-1β induced *MMP1*, *MMP3*, *MMP9*, *MMP13*, *TIMP-1*, *ADAMTS4*, and *ADAMTS5* gene expression. **c** Impact of rhPRG4 treatment in IL-1β-stimulated OA FLS. rhPRG4 (200 μg/ml) treatment reduced *MMP1*, MMP3, *MMP9*, *MMP13* and *ADAMTS5* gene expression in IL-1β-stimulated OA FLS. **d** Role of CD44 in modulating the effect of rhPRG4 on *MMP1*, *MMP3*, *MMP9*, *MMP-13*, and *ADAMTS5* expression in IL-1β-stimulated OA FLS. CD44 neutralization using a CD44 monoclonal antibody (*CD44 Ab*) significantly inhibited the effect of rhPRG4 treatment on *MMP9*, *MMP13*, and *ADAMTS5* expression

rhPRG4 (100 μg/ml) did not significantly alter basal expression of MMP-1, MMP-2, MMP-3, MMP-9, MMP-13, TIMP-1, TIMP-2, ADAMTS4, or ADAMTS5 (Fig. 6a). rhPRG4 (200 μg/ml) significantly reduced basal expression of MMP-1, MMP-3, and MMP-13 (*p* < 0.001). The magnitude of reduction in gene expression was approximately 45% for MMP-1 compared with 58% for MMP-3 and 62% for MMP-13. rhPRG4 (200 μg/ml) significantly increased basal expression of TIMP-2 (*p* < 0.001) (~50% increase) and did not alter the expression of MMP-2, TIMP-1, ADAMTS4, or ADAMTS5.

IL-1β significantly induced *MMP-1*, *MMP-3*, *MMP-9*, *MMP-13*, *ADAMTS4*, and *ADAMTS5* gene expression in OA FLS (*p* < 0.001) (Fig. 6b). rhPRG4 (100 μg/ml) treatment significantly reduced ADAMTS5 expression in IL-1β-stimulated OA FLS (*p* < 0.001) (~35% reduction) (Fig. 6c). rhPRG4 (200 μg/ml) treatment significantly reduced *MMP-1*, *MMP-3*, *MMP-9*, *MMP-13*, and *ADAMTS5* expression in IL-1β-stimulated OA FLS (*p* < 0.001). The magnitude of reduction in gene expression was approximately 51% for *MMP-1* compared with 76% for *MMP-3*, 49% for *MMP-9*, 77% for *MMP-13*, and 60% for *ADAMTS5*. rhPRG4 (100 or 200 μg/ml) treatment did not alter *ADAMTS4* expression. Expression of *MMP-9*, *MMP-13* and *ADAMTS5* genes was significantly higher in the IL-1β + rhPRG4 + CD44 Ab group compared to the IL-1β + rhPRG4 group (*p* < 0.001) (Fig. 6d). In contrast, there was no significant difference in fold expression of MMP-1 or MMP-3 between the IL-1β + rhPRG4 + CD44 Ab group and the IL-1β + rhPRG4 group. Additionally, there was no significant difference in fold expression of *MMP-1*, *MMP-3* or *MMP-13* between the IL-1β + rhPRG4 + CD44 Ab group and the IL-1β group.

### Expression of OA-associated inflammatory cytokines and mediators is affected by rhPRG4 in unstimulated and IL-1β-stimulated OA FLS

**Fig. 7:**
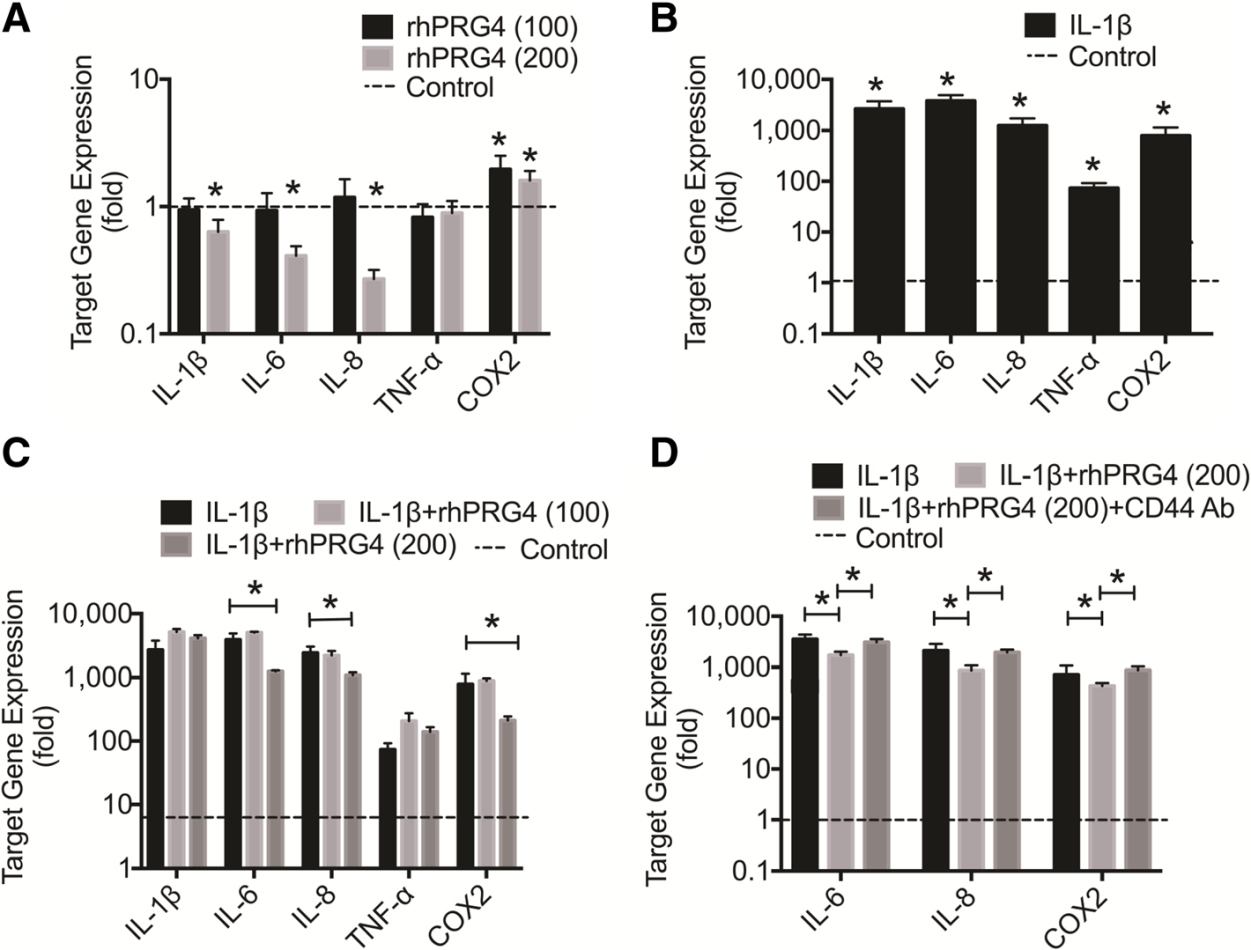
The impact of recombinant human proteoglycan-4 (*rhPRG4*) treatment on basal and IL-1β-induced gene expression of *IL-1*β, *IL-6*, *IL-8*, *TNF-*α, and cyclooxygenase-2 (*COX2*) in osteoarthritis fibroblast-like synoviocytes (OA FLS). Data are presented as the average ± standard error of the mean of four independent experiments. Data are presented as fold change compared to untreated control OA FLS; *p < 0.001. **a** Effect of rhPRG4 treatment on basal gene expression in OA FLS. rhPRG4 (100 μg/ml and 200 μg/ml) increased basal *COX2* expression. rhPRG4 (200 μg/ml) reduced basal *IL-1*β, *IL-6*, and *IL-8* expression. **b** Impact of IL-1β treatment on pro-inflammatory cytokines and mediators of gene expression in OA FLS. IL-1β induced *IL-1*β, *IL-6*, *IL-8*, *TNF-*α, and *COX2* gene expression. **c** Impact of rhPRG4 treatment in IL-1β-stimulated OA FLS. rhPRG4 (200 μg/ml) treatment reduced *IL-1*β, *IL-6*, *IL-8*, *TNF-*α, and *COX2* gene expression in IL-1β-stimulated OA FLS. **d** Role of CD44 in modulating the effect of rhPRG4 on *IL-6*, *IL-8*, and *COX2* expression in IL-1β-stimulated OA FLS. CD44 neutralization using a CD44 monoclonal antibody (*CD44 Ab*) significantly inhibited the effect of rhPRG4 treatment on *IL-6*, *IL-8*, and *COX2* expression

rhPRG4 (100 μg/ml) did not significantly alter basal expression of *IL-1*β, *IL-6*, *IL-8*, *TNF-*α, and significantly increased basal *COX2* gene expression (*p* < 0.001) (~96% increase) (Fig. 7a). rhPRG4 (200 μg/ml) significantly reduced basal expression of *IL-1*β, *IL-6* and *IL-*8 (*p* < 0.001) (~37%, 59%, and 73% decrease) and significantly increased basal expression of *COX2* (~61% increase) (*p* < 0.001). rhPRG4 (100 or 200 μg/ml) did not alter basal expression of TNF-α.

IL-1β significantly induced *IL-1*β, *IL-6*, *IL-8*, *TNF-*α, and *COX2* gene expression (*p* < 0.001) (Fig. 7b). There was no significant difference in *IL-1*β, *IL-6*, *IL-8*, *TNF-*α, or *COX2* gene expression between the IL-1β + rhPRG4 (100 μg/ml) group and the IL-1β group (Fig. 7c). rhPRG4 (200 μg/ml) treatment significantly reduced *IL-6*, *IL-8*, and *COX2* gene expression in IL-1β-stimulated OA FLS (*p* < 0.001). The magnitude of reduction in gene expression was approximately 67% for *IL-6* compared with 55% for *IL-8* and 73% for *COX2*. Expression of *IL-6*, *IL-8* and *COX-2* genes was significantly higher in the IL-1β + rhPRG4 + CD44 Ab group compared to the IL-1β + rhPRG4 group (*p* < 0.001) (Fig. 7d). Finally, there was no significant difference in fold expression of *IL-6*, *IL-8*, or *COX-2* between the IL-1β + rhPRG4 + CD44 Ab group and the IL-1β group.

## Discussion

In this work, we have shown that nuclear levels of NFκB p50 and p65 in *Prg4* ^-/-^ synoviocytes were higher compared to *Prg4* ^+/+^ synoviocytes. Additionally, *Prg4* ^*+/+*^ and *Prg4* ^*-/-*^ synoviocytes had similar levels of p65 subunits in the cytosol while the latter had elevated cytosolic p50 subunit levels. Enhanced nuclear localization of NFκB in *Prg4* ^-/-^ synoviocytes may explain their pro-inflammatory and proliferative capacity under unstimulated conditions and in response to inflammatory stimuli compared to their wildtype counterparts [15]. rhPRG4 inhibited NFκB p50 and p65 nuclear translocation in *Prg4* ^-/-^ synoviocytes and *Prg4* ^*+/+*^ synoviocytes. Interestingly, rhPRG4 treatment reduced cytosolic levels of p50 and p65 proteins in *Prg4* ^*+/+*^ and *Prg4* ^*-/-*^ synoviocytes. This is a unique biological effect of rhPRG4 and may indicate that rhPRG4 functions to reduce the total cellular pool of NFκB, accounting for its anti-proliferative and anti-inflammatory activities. The observed effect of rhPRG4 in relation to nuclear NFκB translocation in murine synoviocytes may not be entirely due to its binding to CD44 receptor as neutralization of CD44 did not completely abolish the effect of rhPRG4.

OA synovitis is characterized by synovial hyperplasia with an increased number of synovial lining cells, neovascularization and inflammatory cell infiltration [26–31]. Patients with OA display a heterogeneous array of synovial pathologic changes and the severity of OA synovitis is correlated with pain and disease progression [32–35]. OA synoviocytes proliferate in response to IL-1 as shown by us and others [36]. As expected, the degree of OA synoviocyte proliferation in our experiment was markedly reduced compared to RA synoviocyte proliferation under similar conditions [15, 36]. rhPRG4 dose-dependently reduced IL-1-induced OA synovial fibroblast proliferation, mediated by inhibition of NFκB p50 and p65 nuclear translocation, in a CD44-dependent manner. The biological effect of rhPRG4 is mediated by its ability to inhibit IκBα phosphorylation subsequent to IL-1 receptor stimulation. IκBα binds to NFκB subunits causing their cytoplasmic retention [37]. Phosphorylation of IκBα results in proteasome-mediated degradation [37]. rhPRG4 prevents IκBα degradation in a CD44-dependent manner; this is consistent with its ability to inhibit IL-1-induced NFκB nuclear translocation. This observation extends the efficacy of rhPRG4 as a biologic agent that inhibits synoviocyte proliferation in RA and OA, and establishes a role for CD44 in modulating OA synoviocyte proliferation [38]. The role of PRG4 in modulating OA synoviocyte proliferation is further highlighted by the ability of PRG4 in SF to reduce OA synoviocyte proliferation. Depleting PRG4 in OA SF enhanced the proliferative effect of IL-1 on OA synoviocytes. PRG4 acts in an autocrine manner to inhibit OA synoviocyte proliferation. This effect is manifested by the enhanced basal and IL-1-induced OA synoviocyte proliferation in response to PRG4 knockdown. This autocrine role for PRG4 validates the synovial hyperplasia observed in *Prg4* ^-/-^ mice and the partial regression of synovial hyperplasia upon Prg4 re-expression [5, 8].

*PRG4* expression by articular chondrocytes and synovial fibroblasts is modulated by mechanical and biological stimuli. Shear upregulates *PRG4* gene expression in articular cartilage [39, 40] and intermittent hydrostatic pressure application enhances PRG4 expression in isolated rat mandibular fibroblasts [41]. Cartilage biosynthesis of PRG4 is reduced as a result of IL-1β and TNF-α exposure but is increased with TGF-β [42–44]. In synoviocytes, *PRG4* expression is reduced by IL-1β and is increased by TGF-β and TGF-β-linked PRG4 accumulation is counterbalanced by IL-1β [42, 45]. In our study, IL-1β and TNF-α reduced PRG4 production in OA synoviocytes but did not reduce PRG4 production in RA synoviocytes. Correspondingly, TGF-β enhanced PRG4 production in OA synoviocytes but did not enhance it in RA synoviocytes. These differences in synoviocyte production of PRG4 extends to basal conditions where OA synoviocytes produced PRG4 to a greater extent compared to RA synoviocytes. While the underlying mechanism of differential production of PRG4 by synoviocytes from different disease origins remains to be elucidated, these phenotypic differences may contribute to the enhanced proliferation and migration seen in RA synoviocytes compared to OA synoviocytes [36].

rhPRG4 exhibited a bimodal concentration-dependent response related to its ability to modulate PRG4 expression in IL-1β-stimulated OA synoviocytes. While rhPRG4 at a low concentration restored *PRG4* expression in OA synoviocytes following IL-1β challenge, rhPRG4 at a higher concentration did not antagonize IL-1β downregulation of PRG4 in OA synoviocytes. Moreover, exogenous PRG4 appeared to directly regulate *PRG4* production by OA synoviocytes. At a low concentration, rhPRG4 treatment did not change basal expression of *PRG4* in OA synoviocytes. Alternatively, exogenous PRG4 exhibited negative feedback on *PRG4* expression in OA synoviocytes as rhPRG4 treatment downregulated basal *PRG4* expression in OA synoviocytes. Our observations extend the understanding of how endogenous PRG4 expression is modulated by exogenous PRG4 in articular cartilage and in in vivo models of posttraumatic arthritis [14, 46].

Synoviocytes play an important role in driving OA pathogenesis. OA synoviocytes express a wide-range of matrix-degrading catabolic enzymes and cytokines. In our study, and in response to IL-1β stimulation, OA synoviocytes induced the expression of *MMP-1*, *MMP-3*, *MMP-9*, *MMP-13*, *TIMP-1*, *aggrecanase-1*, *aggrecanase-2*, *IL-1*β, *TNF-*α, *IL-6*, *IL-8*, and *COX2*. Our results are in agreement with previous studies that reported on the effect of IL-1β on OA synoviocytes [47, 48]. rhPRG4 dose-dependently reduced the expression of *MMP-1*, *MMP-3*, *MMP-9*, *MMP-13*, *aggrecanase-2*, *IL-6*, *IL-8*, and *COX2* in IL-1β-stimulated OA synoviocytes. This anti-inflammatory effect of rhPRG4 was partially mediated by the CD44 receptor, as blocking this receptor abolished the effect of rhPRG4 on *MMP-9*, *MMP-13*, *aggrecanase-2*, *IL-6*, *IL-8*, and *COX2* expression.

rhPRG4 dose-dependently downregulated basal expression of OA-associated enzymes and cytokines. Specifically, rhPRG4 downregulated the expression of *MMP-1*, *MMP-3*, *MMP-9*, and *MMP-13*, which are involved in synovial fibroblast proliferation and migration [49, 50] and cartilage destruction. Interestingly, rhPRG4 reduced the expression of *COX2* in IL-1β-stimulated OA synoviocytes, whereas rhPRG4 marginally increased the expression of COX2 in unstimulated OA synoviocytes. The significance of this observation in the overall biological effect of rhPRG4 remains to be elucidated.

In summary, we herein report on the biological effect of PRG4 on fibroblast-like synoviocytes derived from patients with OA. To our knowledge, this is the first report that delineates the biological role of PRG4 in regulating OA synoviocyte function. Our study adds to existing literature that describes a non-mechanical cartilage-protective effect of PRG4 through upregulation of hypoxia-inducible factor (HIF3α) [51]. An interesting finding in our work is the role of CD44 in modulating the biological activity of rhPRG4 in synoviocytes from different origins. While rhPRG4 functioned to reduce basal NFκB nuclear levels in murine synoviocytes, this effect did not appear to be mediated by the CD44 receptor. Furthermore, rhPRG4 had equal efficacy in *Prg4* ^*-/-*^ synoviocytes and *Prg4* ^*+/+*^ synoviocytes, despite the fact that *Prg4* ^*-/-*^ synoviocytes display enhanced CD44 protein expression compared to *Prg4* ^*+/+*^ synoviocytes [15]. The CD44-dependent activity of rhPRG4 is clearly evident in OA FLS following stimulation with IL-1. In this setting, rhPRG4 inhibited IL-1-induced NFκB activation and downstream expression of catabolic enzymes and inflammatory cytokines in a CD44-dependent manner.

Our findings may be limited by the small number of OA SF aspirates included in the study. Additionally, we have not studied the biological effect of rhPRG4 on FLS from patients with varying degrees of synovitis. A third limitation of our study is that we cannot exclude the possibility that other SF proteins were co-precipitated in the PRG4 immunoprecipitation experiments.

## Conclusion

PRG4 is a glycoprotein secreted by synovial fibroblasts that regulates basal and IL-1-induced expression of matrix metalloproteinases that are involved in synovial proliferation and cartilage destruction. The full-length recombinant form of PRG4 inhibits proliferation of synovial fibroblasts through a mechanism that involves the CD44 receptor. Additionally, depletion of native PRG4 in OA SF stimulates synoviocyte proliferation.

## Abbreviations

Ab: Antibody
ADAMTS: A disintegrin and metalloproteinase with thrombospondin motifs
ANOVA: Analysis of variance
BSA: Bovine serum albumin
COX2: Cycloxygenase-2
Ct: Cycle threshold
DMEM: Dulbecco’s modified Eagle’s medium
ELISA: Enzyme-linked immunosorbent assay
FBS: Fetal bovine serum
FLS: Fibroblast-like synoviocytes
GAPDH: Glyceraldehyde-3-phosphate dehydrogenase
IL-1β: Interleukin-1 beta
MMP: Matrix metalloproteinase
NC: Negative control
NFκB: Nuclear factor kappa B
OA: Osteoarthritis
PBS: Phosphate-buffered saline
p-IκBα: Phosphorylated inhibitor kappa B alpha
PRG4: Proteoglycan-4
qPCR: Quantitative PCR
RA: Rheumatoid arthritis
rhPRG4: Recombinant human PRG4
SF: Synovial fluid
siRNA: Small interfering RNA
TBS-T: Tris-buffered saline Tween 20
TGF-β: Transforming growth factor beta
TIMP: Tissue inhibitor of metalloproteinases
TNFα: Tumor necrosis factor alpha
